# Assessment of Fatty Liver Syndrome and Its Predisposing Factors in a Dairy Herd from Venezuela

**DOI:** 10.1155/2013/191708

**Published:** 2013-04-27

**Authors:** Clara I. Gonzalez, Oswaldo Rosendo

**Affiliations:** Departament of Animal Nutrition and Forages, Veterinary Sciences, University Centroccidental Lisandro Alvarado, Tarabana 3001, Venezuela

## Abstract

The present on-farm research evaluated the occurrence of fatty liver syndrome and its predisposing risk factors for multiparous dairy cows from a commercial herd in Venezuela. Liver biopsy samples were collected at 35 days (d) prepartum (Holstein, *n* = 14; Holstein × Carora crossbred, *n* = 17) as well as 1 to 7 d (Holstein, *n* = 8; Holstein × Carora crossbred, *n* = 11) and 28 to 35 d (Holstein, *n* = 6; Holstein × Carora crossbred, *n* = 14) postpartum in order to analyse hepatic triacylglycerols (TAG, % wet basis) and glycogen concentrations. At postpartum, an occurrence of 72.0% for severe fatty liver along with 73.5% of subclinical ketosis (SCK) was found. The multiple regression model that best explained the association between milk production in the previous lactation (MYP) and TAG at first week postpartum was as follows: TAG, % = −11.2 + 3.16 (prepartum body condition) + 0.0009176 (MYP) (R^²^ = 0.36, *P* < 0.05). Logistic regression indicated that Holstein × Carora crossbred cows tended to have 27% higher relative risk than Holstein to experience SCK, whereas prepartum liver TAG greater than 3% tended to be associated with a higher relative risk for SCK compared to cows with TAG ≤3%.

## 1. Introduction

Fatty liver (hepatic lipidosis) and ketosis (elevated concentrations of ketone bodies in blood, milk, urine, and other body fluids) are metabolic alterations that occur in high producing dairy cows during the periparturient period. Although distinctly different, they are both part of a generalized lipid mobilization syndrome named fatty liver syndrome (FLS), severe fatty liver, or fat cow syndrome. Therefore, FLS is characterized by a high hepatic triacylglycerols infiltration and the subsequent ketosis development associated with retained fetal membranes and metritis at early lactation [1–3]. 

Dry matter intake reductions along with high energy demand for milk production are the main risk factors for FLS [4]. Fatty liver syndrome developed in cows with higher milk yield but quantitative relationships have not been defined. Overconditioning of dairy cows during the dry period predisposes to the development of hepatic lipidosis after calving, due to increased plasma levels of nonesterified fatty acids from lipolysis [5–7]. 

Another factor that could predispose to the development of FLS would be the length of dry period as stated by McCormack [8] but, at farm level, this and other possible risk factors are still poorly understood due in part to the invasive nature of the diagnostic technique for hepatic lipidosis. Moreover, the knowledge of the occurrence of FLS in herds from Venezuela is very limited. Therefore, this research was aimed at evaluating the occurrence of both fatty liver and subclinical ketosis in dairy cows from a commercial herd in Venezuela. A second objective was to determine the associations among chemical composition of the liver, subclinical ketosis, breed, milk production, body condition prepartum, and length of dry period. 

## 2. Materials and Methods 

### 2.1. Management of Cows

The present study was conducted in an intensive commercial dairy unit (2000 lactating cows), located in Quibor, Lara State, Venezuela. This is a dry forest premountain area, which has an average temperature of 25.4°C, a relative humidity of 69%, and an average annual rainfall of 485.5 mm. Forty multiparous (2 to 7 lactations) pregnant cows were randomly selected from a group of 60 to be sampled by liver biopsy at 35 days (d) prepartum as well as 1 to 7 d, and 28 to 35 d postpartum. However, due to logistic problems only a total of 31 (Holstein, *n* = 14 and Holstein × Carora crossbred, *n* = 17), 19 (Holstein, *n* = 8 and Holstein × Carora crossbred, *n* = 11), and 20 (Holstein, *n* = 6 and Holstein × Carora crossbred, *n* = 14) cows were actually sampled at prepartum, 1st week, and 5th week postpartum, respectively. Both during the dry period and after calving, cows were loosed housed. All cows were fed from 30 to 35 days before calving date with a total mixed ration (Table 1). During the first three weeks postpartum cows were fed a total mixed ration for lactating cows (Table 1). Both diets were formulated by a nutritionist consultant at the farm. 

During the study, each ration was sampled twice for chemical analysis: moisture, ash, ether extract (EE) and crude protein (CP) (the Kjeldahl method) [10], neutral detergent fiber [11], and acid detergent fiber [10]. Lignin, neutral detergent insoluble crude protein (NDICP), and acid detergent insoluble crude protein (ADICP) were assessed by the procedure of Lo et al. [12]. NE_L_ concentrations (Mcal/kg DM) were calculated from proximal composition using the summative equations proposed by NRC [9].

### 2.2. Sampling and Chemical Analyses

Body condition scores (BCS) were measured prepartum and during the first, third, and fifth weeks postpartum, using the scale of 1 to 5 points with a range of 0.25 units [13]. The weight of each cow was determined five weeks before calving and the first, third, and fifth weeks postpartum, using a weigh tape to measure the size of the chest. Liver biopsy samples were collected with cannula and trocar following the procedure described by Rosendo and Mcdowell [14]. The liver samples were placed on filter paper to remove excess blood before placing into cryogenic tubes (Corning tubes, Fisher) and immediately frozen in liquid nitrogen. Samples were stored at −70°C until analyzed. Liver samples were hydrolyzed in a mixture of ethanol and potassium hydroxide, and then triacylglycerol concentrations were determined in the supernatant by using an enzyme kit (Qualitest, Qualitest Industries, Caracas, Venezuela). Liver triacylglycerol content was expressed as percentage of fresh liver TAG. The liver was considered normal when the concentration of liver TAG was less than 1%, mild fatty when the levels were between 1% and 3%, moderate fatty when the liver TAG levels were between 3% and 5%, and severe fatty when the liver TAG concentration exceeds 5% (wet basis) according to the criterion of Bauchart et al. [15]. Liver glycogen (GLY) concentration was measured in the precipitate obtained from the liver hydrolysis, following the method of Lo et al. [12]. 

The detection of subclinical ketosis was achieved in a total of 34 cows in the first, third, and fifth weeks postpartum by the reaction of a sample of fresh milk with sodium nitroprusiato (Ketotest Elanco Animal Health) to detect levels of *β*-hydroxybutyrate in a semiquantitative measurement scale. Positive cows were those that have a color scale corresponding to milk *β*-hydroxybutyrate concentrations ≥200 *μ*mol/L whereas negative cows were those with no reaction to the test or those whose color scale corresponded to milk *β*-hydroxybutyrate concentrations ≤100 *μ*mol/L [16]. 

### 2.3. Statistical Analysis

Information on production records was collected from the farm, retrospectively. Total milk yield, both in the current and the previous lactations, was adjusted to 305 days. The length of dry period (number of days during dry period) was calculated for each cow. Therefore, the following performance parameters were available: prepartum body condition (PBC), body condition at calving (BCC), milk production in the previous lactation (MYP), dry period length (DPL), and number of lactations (NLAC). Also information was collected on the events of mastitis, metritis, rebreeding, foot problems, and deaths. Associations between milk yield in previous lactation (MYP), PBC, and liver TAG or glycogen concentrations were examined by Pearson's correlation using PROC CORR in SAS [17]. The association between the concentration of TAG or liver glycogen in the first week postpartum (dependent variable) and liver glycogen concentration at prepartum (GLYP), liver TAG concentration at prepartum (TAGP), and performance parameters as independent variables was evaluated through multiple linear regressions using PROC GLM [17]. A simple analysis of variance was conducted to determine the effect of the length of dry period (<100 d, short length = SL, and >100 d, large length = LL) on the concentration of TAG or liver glycogen by using PROC GLM of SAS [17].

Factors affecting SCK were analyzed using logistic regression by PROC GENMOND of SAS [17] under the Poisson distribution with the REPEATED statement. The cow was used as subjective as the employment covariance matrix is an unstructured covariance (UNSTR). After evaluating the association between SCK and the potential independent variables (body condition, milk production, liver TAG or liver glycogen concentration, loss of weight or body condition, length of dry period, and breed), a final model was built up with the variables that tended to significance (*P* ≤ 0.15) in the statistical score analysis type 3 GEE (generalized estimation equation). At the end, the following independent variables remained in the model: breed (Holstein = 0, Holstein × Carora crossbred = 1) and liver TAG concentration at prepartum (≤3.0% = low, >3.0% = high). The relative risks were calculated instead of odds ratios by using the ESTIMATE option. The relative risks compare the likelihood of success or failure in each group at the level of the independent variable under investigation. By using PROC GLM of SAS [17], an analysis of variance was performed to determine the main effects of liver TAG accumulation (≤5% and >5%, severe fatty liver) and breed, as well as their interaction in milk production during current lactation, prepartum live weight, prepartum body condition, and dry period length. Statistical significant differences were declared at 95% level, but a tendency toward being significant was considered at *P* = 0.05 to *P* = 0.15. 

## 3. Results and Discussion 

### 3.1. Occurrences of Fatty Liver and Subclinical Ketosis

The average concentration of liver TAG (fresh basis) five weeks before calving (*n* = 31) was 2.9% (1.8 to 6.9%, SE = 1.3). At the first week postpartum (*n* = 19), the average was 6.1% (3.1 to 11.8%, SE = 2.5), while in the fifth week postpartum (*n* = 20), the average liver TAG concentration reached 6.9% (1.3 to 12.3%, SE = 3.6). The mean concentration of liver glycogen (fresh basis) five weeks before calving (*n* = 31) was 3.6% (0.6% to 6.6%, SE = 1.6). For the first week postpartum (*n* = 19), the average reached 1.2% (0.2 to 3.9%, SE = 1.1). Finally, at the fifth week postpartum (*n* = 15), the average liver glycogen concentration was 2.7% (0.6% to 5%, SE = 1.3). All liver TAG and glycogen concentrations had a normal distribution (Shapiro-Wilk, *P* > 0.05). 

In the present study, most lactating cows (72%, Table 2) were classified as severe fatty liver (fresh liver TAG > 5%) according to the scale proposed by Bauchart et al. [15]. This criterion seems appropriate to include all disorders observed at the present on-farm study as FLS. Similar occurrences have been reported in Europe, United States, and Iran [18–20] when fatty liver is defined on the same basis (>5% of TAG, fresh basis), which means that, if risk factors are kept around the year, a total of 1400 lactating dairy cows would exhibit FLS at this farm. 

The high occurrence of FLS in this study is concomitant with very large dry periods (>100 d) (Table 3), which might be due to low postpartum energy intake during short lactations followed by an accumulative high intake of energy during the dry period as well [4, 21]. In the present study, the levels of postpartum diet energy were 10% lower than those recommended (1.60 NEL Mcal/kg DM) for cows with milk production of 30 kg/d or higher [9]. Low levels of diet energy during the postpartum period reduced the capacity of liver to oxidize long chain fatty acids [22]. Therefore, fatty liver is linked to both a high prepartum energy intake as well as to low postpartum energy intake, although immediate feeding during postpartum is probably the most critical for the transition cow as suggested by Grummer [23].

In retrospect, there were no differences in prepartum body weight between cows with or without fatty liver (Table 3). However, overconditioning or obesity near calving is one of the major risk factors for liver TAG infiltration [24, 25]. In the prepartum, the rate of obesity observed in this study (BCS > 4.0) was 47%, well above the 10% accepted as normal [26, 27]. Also, the occurrence of mild fatty liver (1–3% of TAG, fresh basis) reached 71.0% at prepartum according to the criterion of Bauchart et al. [15]. These findings are consistent with those reported by Gerloff et al. [18]. They found a significant accumulation of TAG in the liver during the last 3 weeks prepartum in cows that ended up with severe fatty liver during postpartum.

In the present study, severe postpartum fatty liver was present in Holstein cows associated with a higher prepartum body condition (PBC) as compared to Holstein × Carora cows (4.21 versus 3.70, *P* = 0.11) (Table 3). Gerloff et al. [18] also reported that cows with severe fatty liver had a higher PBC (3.29 versus 2.97) than normal cows but mean BCS were notably lower than in the current study. At the first week postpartum, Rukkwamsuk et al. [7] found higher liver concentrations of TAG on those cows with greater PBC. In the present study, however, no correlation (*P* > 0.15) was found between PBC and liver TAG at first week postpartum. Obese cows have an increased adipose tissue lipolysis and a decreased food intake during the peripartum, which predisposes to the development of fatty liver [7].

For recording the incidence of SCK (new cases), ketotest results above 200 uM of milk *β*-hydroxybutyrate were considered positive, Rosendo et al. [16]. Among all cows sampled, 25 (73.5%) were positive to SCK, of which 8 were Holstein and 17 were crossbred (Holstein × Carora) cows (data not shown in tables). Nine cows were negative (Holstein, *n* = 6; Holstein × Carora, *n* = 3). The incidence of SCK in the first nine weeks of lactation varies from 9 to 34% as reviewed by Rosendo et al. [16]. The high incidence of SCK in the present study was associated with the development of fatty liver, both pre- and postpartum, as well as with several cases of mastitis and metritis (Table 4). The risk of experiencing SCK has been associated with a high body condition (≥3.5) at calving [28]. 

### 3.2. Relationship between Breed, Liver TAG Concentration, and Subclinical Ketosis

The relationship between SCK, breed, and prepartum liver TAG levels was analyzed by logistic regression (Table 5). Crossbred cows (Holstein × Carora) were associated with an increased relative risk, 27% more than Holstein to experience SCK. Some studies have showed differences to the susceptibility for clinical ketosis among breeds. Thus, Holstein cows had a relative risk, 57% higher than Ayrshire or Guernsey to experience clinical ketosis [29]. Although limited by the number of observations, the current study is the first to analyze the susceptibility of Holstein × Carora crossbred cows for developing SCK. Moreover, this study is the first to analyze the prepartum level of hepatic infiltration as a risk factor in the development of SCK. Thus, prepartum hepatic TAG infiltration greater than 3% was associated with an increased relative risk for SCK, 17% more than cows with a liver TAG concentration ≤3%. Several studies found that fatty liver precedes the development of ketosis [25, 30, 31] but no quantitative relationship was established.

### 3.3. Association between Milk Production and the Liver TAG or GLY Concentration during the Peripartum

The analysis showed that fatty liver (TAG > 5%, fresh basis) was present in those cows that produced more milk (6841.5 versus 4395.7 kg, Table 3) during the current lactation. These findings confirm old studies from Europe; 5267 versus 4407 [32] and 6094 versus 5527 [33] kg of milk for cows with fatty versus normal liver but not a recent study [34], in which comparable milk yields were observed among cows with high and low liver fat accumulation after calving.

At the present study, no associations were found between milk production during the current lactation (MYC) and liver TAG concentrations during the first and fifth weeks postpartum (*P* > 0.15) or with liver glycogen concentration at the first week postpartum (*P* > 0.15). But on the other hand, MYC was negatively associated with liver glycogen concentration at the fifth week postpartum (*r* = −0.54, *P* < 0.05). 

The milk production in the previous lactation (MYP) tended to be positively associated with liver TAG concentration during both the first and fifth weeks postpartum (*r* = 0.36 for both correlations, *P* < 0.15) and tended to be negatively associated with the concentration of liver GLY at the fifth week postpartum (*r* = −0.40, *P* < 0.15). None associations were found (*P* > 0.15) between MYP and liver GLY concentration at prepartum or at first week postpartum, as well as between MYP and liver TAG concentration at prepartum. The multiple regression model that best explained the association between MYP and the liver TAG concentration at first week postpartum was as follows: liver TAG concentration = −11.2 + 3.16 (PBC) + 0.0009176 (MYP) (*R* ^2^ = 0.36, *P* < 0.05). 

Results suggest that FLS developed in those cows with a greater potential to produce milk but the association between lactation performance and fatty liver was not clear. As discussed by Ingvartsen et al. [35], there are many other biological correlations that confound the relationship between metabolic diseases such as ketosis and milk production. Rather than the milk yield per se, the causes of production diseases should be sought in the physiological imbalance that occurs as a result of a rapid postcalving acceleration in milk yield [35]. On the other hand, the rapid liver glycolysis may stimulate milk lactose secretion. As lactose secretion constitutes the major driving force of milk water secretion, its increment induces milk yield, and therefore an increase in milk yield correlates with a decrease in liver GLY.

The present study found a negative correlation between liver TAG and liver GLY concentrations at the first week postpartum (*r* = −0.51, *P* < 0.05). Others have found similar results (*r* = −0.72 and −0.39) [36, 37].

### 3.4. Influence of Dry Period Length on the Liver TAG or GLY Concentration during the Peripartum

In this study, the length of dry period discriminated as short length (<100 d, = SL) and large length (>100 d, = LL) did not affect the liver concentrations of TAG or GLY (*P* > 0.15) (Table 6). Moreover, the length of dry period was higher than 100 d in normal and in fatty liver cows as well (Table 3). Some authors have suggested that prolonged dry periods are associated with an increased risk for developing FLS [8], but there is no quantitative information. Pioneers studies found no difference in the incidence of ketosis, milk fever, and retained placenta when comparing different dry periods lengths, 20, 30, 40, 50, and 60 days [38], or even greater, 4, 7, and 10 weeks [39]. Recent studies about the effect of short dry periods on plasma NEFA concentrations do not allow to make precise the number of dry days that is really associated with FLS [40–42]. However, a very short dry period (34 days) could reduce the risk of developing fatty liver, due to lower NEFA concentrations compared with longer dry periods (55 days), as shown by Watters et al. [42]. 

## 4. Conclusions

The present on-farm research revealed a high occurrence of prepartum mild fatty liver, accompanied by a high occurrence of severe fatty liver, up to 72%, and an extremely high incidence of subclinical ketosis during the postpartum period. There was a high percentage of prepartum obesity even though the level of energy for the prepartum ration was below that recommended by the NRC. Fatty liver syndrome developed in those cows with a greater potential to produce milk but associations between lactation performance and fatty liver are still unclear. Crossbred cows, Holstein × Carora, tended to have a higher relative risk (27% more than Holstein cows) to experience subclinical ketosis. Therefore, the study suggests breed differences in the susceptibility to both fatty liver and SCK that deserve further study. Based on TAG values greater than 3%, postpartum fatty liver syndrome might be predicted from 35 days before calving.

## Figures and Tables

**Table 1:** Ingredients and nutritional composition of supplied total mixed rations (TMR).

	Prepartum TMR	Postpartum TMR
	% dry basis	
Ingredients		
Corn silage	11.0	20.5
Wheat bran	6.6	—
Corn meal	19.5	14.9
Fish meal	1.1	—
Soybean meal	3.8	9.8
Distiller grains	6.3	8.7
Bermuda hay	25.0	15.1
Citrus pulp	9.2	7.6
Mineral mix	2.7	—
Brewer's yeast	14.2	19.8
Others	3.2	3.6

Chemical composition		
DM	42.0	43.7
OM	91.6	91.7
CP	13.3	16.1
EE	3.1	5.4
NFC*	29.1	33.3
NDF	53.3	43.4
ADF	30.0	22.9
NDICP	7.1	6.5
ADICP	2.6	2.0
NE_L_ Mcal/kg DM**	1.38	1.48

Other: sulfur flower (0.1%), phosphate 18 (prepartum diet = 0.5% and postpartum diet = 0.9%), niacin (prepartum diet = 0.05% and postpartum diet = 0.03%), calcium carbonate (postpartum diet = 0.6%), sodium bicarbonate (postpartum diet = 1.3%), sodium chloride (postpartum diet = 0.5%), sodium monensin (postpartum diet = 0.01%), yeast-sac (postpartum diet = 0.09%), and vitamins (postpartum diet = 0.05%). DM: dry matter, OM: organic matter, CP: crude protein, EE: ether extract, NFC: nonfiber carbohydrates, NDF: neutral detergent fiber, ADF: acid detergent fiber, NDICP: neutral detergent insoluble crude protein, ADICP: acid detergent insoluble crude protein.

*NFC calculated by difference: 100 − [NDF − NDICP] + CP + EE + ash.

**NE_L_ Mcal/kg = (0.703 × ME − 0.19) + [(0.097 × ME + 0.19)/0.97] × (EE − 3) (NRC, 2001 [9]).

**Table 2:** Occurrence of fatty liver and subclinical ketosis during the first five weeks postpartum.

Metabolic disorders	*n*	Frequency, %
Liver TAG^1^, % fresh basis		
≤5	8	27.6
5	21	72.4
Subclinical ketosis^2^		
Positives	25	73.5
First week	14	41.2
Third week	11	32.3
Negatives	9	26.5
Total	**34**	**100.0**

^1^Cows sampled by liver biopsy at 1st or 5th week postpartum. *n*: number of cows. Frequency = (affected cows/total number of sampled cows) × 100. ^2^Subclinical ketosis: positives: color scale that corresponded to milk *β*-hydroxybutyrate concentrations ≥200 *μ*mol/L. Negatives: color scale that corresponded to milk *β*-hydroxybutyrate concentrations ≤100 *μ*mol/L.

**Table 3:** Effect of liver triacylglycerols (TAG) accumulation on milk yield (current lactation), prepartum live weight, and dry period length.

	Liver TAG, % fresh basis		*P*
	≤5% (*n* = 7)	>5% (*n* = 22)	
MYC, kg	4395.7^a^ ± 761.4	6841.5^b^ ± 701.9	<0.03
LWP, kg	756.2 ± 21.8	729.4 ± 12.6	<0.30
DPL, days	113.4 ± 28.3	123.7 ± 17.1	<0.76

*n*: number of cows; MYC: milk production in the current lactation; LWP: prepartum live weight; BCP: prepartum body condition score; DPL: dry period length; H: Holstein, F_1_: Holstein × Carora crossbred.

**Table 4:** Frequency of other problems during the first 100 days postpartum.

Problem	*n*	Frequency, %
Mastitis	6	16.2
Metritis	6	16.2
Rebreeding	4	10.8
Foot/leg problems	1	2.7
Death	4	10.8

*n*: number of cows.

**Table 5:** Relative risks for the association between subclinical ketosis (dependent variable), breed, and prepartum levels of liver triacylglycerols (Liver TAG).

Independent variable	RR	SE	CI	*P*
Breed (0 versus 1)	0.73	0.13	0.52–1.03	<0.08
Liver TAG (H versus L)	1.17	0.11	0.97–1.40	<0.10

RR: relative risk; SE: standard error; CI: confident interval; *χ*²: chi square; breed: 0 = Holstein; 1 = Holstein × Carora crossbred. Liver TAG: H: high, >3.0% TAG, and L: low, ≤3.0% TAG (fresh basis).

**Table 6:** Effect of dry period length on liver concentrations of triacylglycerols (TAG) and glycogen (GLY) in the first week postpartum.

	Liver concentration, % fresh basis	
	TAG	GLY
SL, <100 d	5.7	1.0
LL, >100 d	6.6	1.3
SD	0.9	0.4
*n*	19	19
*P*	0.52	0.55

SL: short length. LL: large length. *n*: numbers of cows. SD: standard deviation. *P*: probability.
